# Clinical Profiles, Interventions, and Outcomes of Sepsis and Septic Shock in a Saudi Arabian Tertiary ICU: A Five-Year Retrospective Analysis

**DOI:** 10.3390/healthcare14050680

**Published:** 2026-03-07

**Authors:** Amer Asiri, Khaled Abdulwahab Amer, Mushary Alqahtani, Lena A. Almathami, Osama Ayed Asiri, Sultan Saad Alnasser, Ahmed Ali Khuzayyim, Bander Abdullah Alqahtani, Fatimah Mohammed Asiri, Hatem Mostafa Asiri

**Affiliations:** 1 Intensive Care Unit, King Khalid University Medical City, Abha 62529, Saudi Arabia; amerasiri80@gmail.com; 2 Department of Internal Medicine, Aseer Central Hospital, Aseer Health Cluster, Abha 62523, Saudi Arabia; fatimahasiri2020@gmail.com; 3 Department of Internal Medicine, Armed Forces Hospital Southern Region, Khamis Mushait 62413, Saudi Arabia; mushary50@gmail.com (M.A.); sultannassir450@gmail.com (S.S.A.); 4 Department of Otolaryngology-Head and Neck Surgery, Armed Forces Hospital Southern Region, Khamis Mushait 62413, Saudi Arabia; lenaalmathami@gmail.com; 5 Department of Internal Medicine, Khamis Mushait General Hospital, Khamis Mushait 62413, Saudi Arabia; ix.vip673@gmail.com; 6 King Abdullah Medical Complex and Maternity and Children Specialized Hospital, Jeddah Second Health Cluster, Jeddah 23513, Saudi Arabia; ahmad3212376@gmail.com; 7 General Practice, Aseer Health Cluster, Abha 62523, Saudi Arabia; bander7703@gmail.com; 8 Department of General Surgery, Armed Forces Hospital Southern Region, Khamis Mushait 62413, Saudi Arabia; hatem14201@gmail.com

**Keywords:** sepsis, septic shock, intensive care unit, mortality, Saudi Arabia, Sepsis-3, urinary tract infection, diabetes mellitus, antimicrobial therapy

## Abstract

**Background and Objectives**: Sepsis and septic shock remain leading causes of morbidity and mortality in intensive care settings worldwide. While substantial epidemiological data exist from Western countries, the clinical profile of sepsis in regions with exceptionally high diabetes prevalence remains inadequately characterized. Saudi Arabia, with one of the highest diabetes mellitus prevalence rates globally, may exhibit distinct sepsis epidemiology, infection patterns, and outcomes. This study aimed to characterize the clinical profiles, antimicrobial management, and outcomes of sepsis and septic shock in a tertiary intensive care unit (ICU) in the Aseer region of southwestern Saudi Arabia. **Materials and Methods**: A retrospective observational study was conducted including 263 adults meeting Sepsis-3 criteria (232 sepsis, 31 septic shock) admitted to a tertiary ICU between January 2020 and December 2024. Demographics, comorbidities, laboratory parameters, microbiological data, antibiotic timing, interventions, and in-hospital mortality were analyzed. Logistic regression identified independent mortality predictors. This study adhered to the STROBE reporting guidelines. **Results**: The median age was 73 years with male predominance (58.4%). Diabetes mellitus (71.5%) and hypertension (65.8%) were highly prevalent. Urinary tract infections (UTIs) predominated (79.8%), with Escherichia coli as the most common pathogen (26.2%). The median time to antibiotic administration was 1.8 h; piperacillin–tazobactam was the most frequent empiric regimen (43.7%). Septic shock patients exhibited higher creatinine (1.65 vs. 1.08 mg/dL, *p* = 0.026) and lower platelets (194 vs. 271 × 10^3^/μL, *p* = 0.030). Mortality was 38.7% in septic shock versus 8.2% in sepsis (*p* < 0.001). Multivariate analysis confirmed septic shock (aOR: 5.23; 95% CI: 1.89–14.48) and mechanical ventilation (aOR: 15.42; 95% CI: 5.67–41.95) as independent mortality predictors. **Conclusions**: High diabetes prevalence shapes regional sepsis epidemiology with UTI predominance. Early antibiotic administration and recognition of septic shock remain critical for improving outcomes in this population.

## 1. Introduction

Sepsis represents a life-threatening organ dysfunction caused by dysregulated host response to infection, while septic shock denotes a subset with profound circulatory and metabolic abnormalities substantially elevating mortality risk [1,2]. The Third International Consensus Definitions (Sepsis-3) have standardized diagnostic criteria, emphasizing Sequential Organ Failure Assessment scoring for identifying infection-related organ dysfunction [1]. Globally, sepsis affects approximately 49 million individuals annually with 11 million deaths, representing nearly 20% of worldwide mortality [3].

The Surviving Sepsis Campaign (SSC), updated in 2021, provides evidence-based management bundles emphasizing early antibiotic administration within one hour for septic shock and three hours for sepsis without shock [4,5]. Recent meta-analyses confirm that delayed antibiotic administration beyond one hour is associated with increased mortality [6]. Despite guideline dissemination, implementation varies considerably across healthcare systems and geographic regions.

Epidemiological patterns of sepsis differ substantially across regions, influenced by prevalent pathogens, antimicrobial resistance profiles, comorbidity burdens, and healthcare infrastructure [7]. Saudi Arabia presents unique epidemiological characteristics given its exceptionally high diabetes mellitus prevalence—among the highest globally [8]. Diabetes predisposes individuals to infections through multiple mechanisms including impaired neutrophil function, glycosuria-promoted bacterial proliferation, and autonomic neuropathy affecting bladder emptying [9]. A recent national multicenter study in Saudi Arabia highlighted the growing sepsis burden within the Kingdom, reporting substantial variation in detection and outcomes across settings [10]. The interplay between the high prevalence of metabolic diseases and sepsis epidemiology in Gulf Cooperation Council countries remains poorly understood, necessitating region-specific investigations.

Limited published data characterize sepsis burden in Saudi Arabian critical care settings. The landmark retrospective cohort study by Arabi et al. from Riyadh reported declining mortality trends over 16 years in a cohort of 5917 patients [11]. However, regional variation within Saudi Arabia remains largely unexplored, particularly in areas with distinct demographic and comorbidity profiles. The Aseer region in southwestern Saudi Arabia serves a predominantly Saudi population with high rates of diabetes and cardiovascular disease, yet no prior study has examined sepsis epidemiology in this setting. Understanding region-specific patterns is essential for tailoring empiric antimicrobial selection, optimizing sepsis bundles, and allocating critical care resources. This investigation aimed to characterize the clinical profiles, infection sources, antimicrobial management, and outcomes of adult patients with sepsis and septic shock at a tertiary intensive care unit (ICU) in southwestern Saudi Arabia, with particular attention to the influence of the regional comorbidity burden on sepsis epidemiology.

We hypothesized that the exceptionally high diabetes prevalence in this population would influence the predominant infection sources, organism profiles, and potentially the response to treatment, distinguishing regional sepsis epidemiology from both national and international patterns.

## 2. Materials and Methods

### 2.1. Study Design and Participants

This retrospective observational study was conducted at the ICU of a tertiary referral hospital in the Aseer region of southwestern Saudi Arabia and reported in accordance with the Strengthening the Reporting of Observational Studies in Epidemiology (STROBE) guidelines (Supplementary File S1) [12]. Medical records spanning 1 January 2020 through 31 December 2024 were systematically reviewed. Adult patients aged ≥18 years meeting Sepsis-3 diagnostic criteria were included [1]. Sepsis was defined as suspected or documented infection accompanied by acute organ dysfunction (Sequential Organ Failure Assessment [SOFA] score increase ≥ 2 points from baseline). Septic shock was identified when patients required vasopressor administration to maintain mean arterial pressure ≥ 65 mmHg despite adequate volume resuscitation, with serum lactate > 2 mmol/L. Time zero for sepsis onset was defined as the earliest documented time at which Sepsis-3 criteria were met, based on clinical documentation of infection suspicion concurrent with SOFA score elevation.

Exclusion criteria comprised the following: (1) incomplete documentation precluding confirmation of Sepsis-3 criteria (n = 42, representing 8.7% of screened admissions), specifically cases lacking documented infection source or SOFA scoring data; (2) transfer from external facilities > 24 h after sepsis onset, as the timing of initial interventions could not be reliably ascertained (n = 18); (3) cases with non-infectious systemic inflammatory response syndrome (SIRS) mimicking sepsis (n = 42); and (4) patients aged <18 years (n = 93). Of 458 screened admissions, 263 patients meeting all inclusion criteria were enrolled (Supplementary File S1). No a priori power calculation was performed given the retrospective design; however, a post hoc power analysis confirmed that the sample of 263 patients provided >80% power to detect an odds ratio of 3.0 for the primary outcome (in-hospital mortality) at α = 0.05, given the observed mortality rate of 11.8%.

### 2.2. Data Collection

Data were extracted using a standardized case report form developed for this study. Demographic variables included age, sex, nationality, and body mass index. Comorbidities were systematically recorded including diabetes mellitus (type 1 or type 2, and antidiabetic medication class: insulin, metformin/biguanides, sodium–glucose co-transporter 2 [SGLT2] inhibitors, and others), hypertension, chronic kidney disease (CKD), coronary artery disease, chronic liver disease, malignancy, chronic obstructive pulmonary disease, cerebrovascular disease, and immunosuppressive conditions. Laboratory parameters comprised peak values during ICU admission for serum creatinine, white blood cell (WBC) count, lactate, glucose, total bilirubin, platelet count, C-reactive protein (CRP), and erythrocyte sedimentation rate (ESR). Peak laboratory values were selected as they represent the maximum physiological derangement during the ICU course; however, a limitation is that these peaks may have occurred at variable time points relative to sepsis diagnosis. Microbiological data included infection source, isolated organisms, and antimicrobial susceptibility patterns including extended-spectrum beta-lactamase (ESBL) production.

Antimicrobial data comprised time from sepsis recognition to first antibiotic dose (door-to-antibiotic time), empiric antibiotic regimen, and appropriateness of empiric coverage based on subsequent culture results. Documented therapeutic interventions included mechanical ventilation, continuous renal replacement therapy (CRRT), extracorporeal membrane oxygenation (ECMO), and vasopressor requirements. ECMO was utilized primarily for acute respiratory distress syndrome (ARDS)-associated refractory hypoxemia rather than for isolated septic shock, consistent with current evidence-based indications [13]. The primary outcome was in-hospital mortality. Secondary outcomes included ICU length of stay and 30-day mortality where available.

### 2.3. Ethical Considerations

This study was approved by the Aseer Institutional Review Board (H-06-B-091), approval number: REC-9–1–2023. Informed consent was waived given the retrospective anonymized design, in accordance with national research regulations and the Declaration of Helsinki. All patient data were de-identified prior to analysis.

### 2.4. Statistical Analysis

Continuous variables are presented as medians with interquartile ranges (IQRs) given predominant non-normal distributions assessed by Shapiro–Wilk testing. Between-group comparisons employed the Mann–Whitney U test. Categorical variables are expressed as frequencies and percentages, with Fisher’s exact test for comparisons. Univariate logistic regression identified potential mortality predictors. Variables with *p* < 0.10 in univariate analysis were entered into multivariate logistic regression using backward stepwise elimination. Results are reported as adjusted odds ratios (aORs) with 95% confidence intervals (CIs). Model fit was assessed using the Hosmer–Lemeshow test. To assess for potential effect modification, the interaction between ARDS and mechanical ventilation was examined. Model internal validation was evaluated through the Hosmer–Lemeshow goodness-of-fit test; however, advanced validation techniques such as bootstrapping or split-sample validation were not performed due to the limited sample size, which is acknowledged as a limitation. Statistical significance was defined at α = 0.05. Analyses were performed using SPSS version 28 (IBM Corp., Armonk, NY, USA).

## 3. Results

### 3.1. Baseline Characteristics

The study cohort comprised 263 patients: 232 (88.2%) with sepsis and 31 (11.8%) with septic shock (Table 1). The median age was 73 years (IQR: 55–84), with 153 participants (58.4%) being male. Saudi nationals constituted 96.2% of the sample. Diabetes mellitus affected 188 patients (71.5%)—notably higher than the 48.3% reported by Arabi et al. [11] and substantially exceeding global estimates of approximately 20% [14]. Hypertension was documented in 173 patients (65.8%). No significant baseline differences existed between groups.

### 3.2. Infection Characteristics and Antimicrobial Management

**Figure 1:**
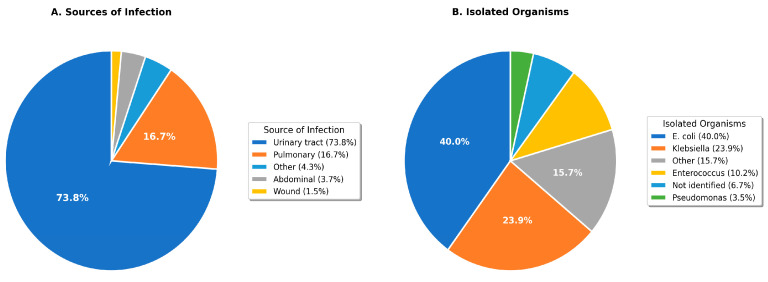
Distribution of infection sources (**A**) and isolated organisms (**B**) among sepsis patients. UTI: urinary tract infection.

Urinary tract infections predominated as the sepsis source, identified in 210 patients (79.8%)—markedly exceeding rates in other Saudi studies (Qassim: 1.6% [15]) and international literature (12–15% globally [3]). Pulmonary infections were identified in 37 patients (14.1%). Escherichia coli was the most frequently isolated organism (69 patients, 26.2%), followed by Klebsiella pneumoniae (45 patients, 17.1%) and Enterococcus species (22 patients, 8.4%). The culture positivity rate was 61.2% (161/263). Extended-spectrum beta-lactamase (ESBL) production was detected in 34.8% of Enterobacteriaceae isolates (Table 2, Figure 1).

Median time from sepsis recognition to first antibiotic administration was 1.8 h (IQR: 1.1–2.9). One-hour bundle compliance (antibiotics within 60 min) was achieved in 38.4% of septic shock patients and 22.8% of sepsis patients. Piperacillin–tazobactam was the most common empiric regimen (115 patients, 43.7%), followed by meropenem (78 patients, 29.7%) and ceftriaxone (42 patients, 16.0%). Empiric antibiotic coverage was appropriate (concordant with culture susceptibilities) in 73.9% of culture-positive cases (Table 3).

### 3.3. Laboratory Findings

**Figure 2:**
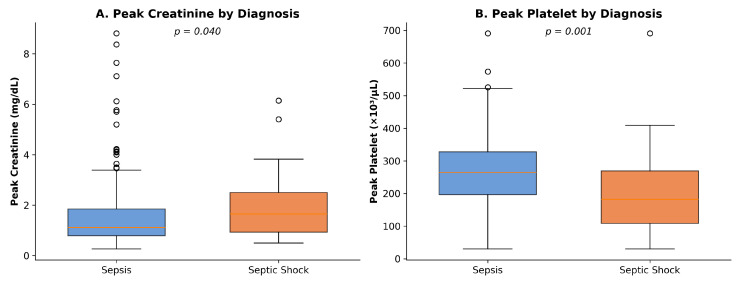
Box plots comparing (**A**) peak creatinine and (**B**) peak platelet counts between sepsis and septic shock groups.

Comparative analysis revealed significant differences between groups (Table 4, Figure 2). Peak serum creatinine was elevated in septic shock (median 1.65 mg/dL, IQR: 0.92–2.50) compared to sepsis (median 1.08 mg/dL, IQR: 0.75–1.81; *p* = 0.026), reflecting acute kidney injury from circulatory compromise. Platelet counts were lower in septic shock (median 194 × 10^3^/μL, IQR: 112–323) versus sepsis (median 271 × 10^3^/μL, IQR: 197–369; *p* = 0.030), consistent with coagulation cascade activation. Serum lactate trended higher in septic shock but did not reach statistical significance (*p* = 0.089).

### 3.4. Interventions and Outcomes

**Figure 3:**
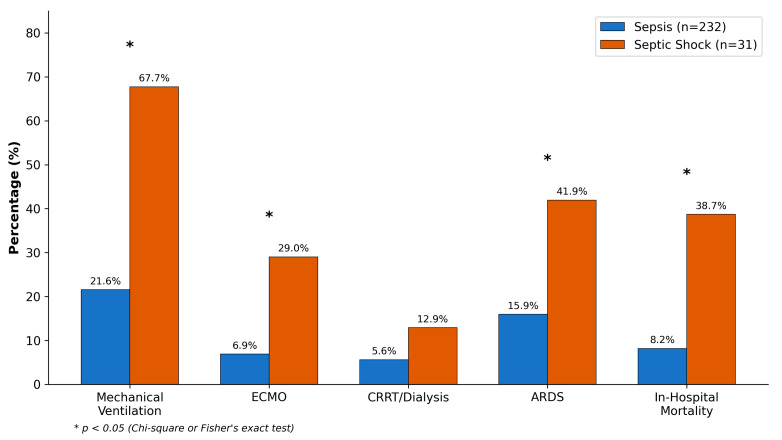
Comparison of interventions and clinical outcomes between sepsis and septic shock groups. * *p* < 0.05.

Organ support requirements differed substantially between groups (Table 5, Figure 3). Mechanical ventilation was instituted in 21 septic shock patients (67.7%) compared to 50 sepsis patients (21.6%; *p* < 0.001). Norepinephrine was the primary vasopressor, used in all septic shock patients at a median maximum dose of 0.25 μg/kg/min (IQR: 0.12–0.48). ECMO, deployed for ARDS-associated refractory hypoxemia, was utilized in 9 septic shock patients (29.0%) and 16 sepsis patients (6.9%; *p* < 0.001). ARDS developed in 41.9% of septic shock versus 16.0% of sepsis patients (*p* < 0.001).

In-hospital mortality was 38.7% (12/31) in septic shock versus 8.2% (19/232) in sepsis (*p* < 0.001). Overall mortality was 11.8% (31/263). Median ICU length of stay was 6 days (IQR: 4–11) overall, with septic shock patients having longer stays (9 days, IQR: 5–16) compared to sepsis (6 days, IQR: 4–10; *p* = 0.018). Thirty-day mortality data, available for 78% of patients, showed similar patterns (septic shock: 45.8%, sepsis: 10.3%).

### 3.5. Mortality Risk Factors

**Figure 4:**
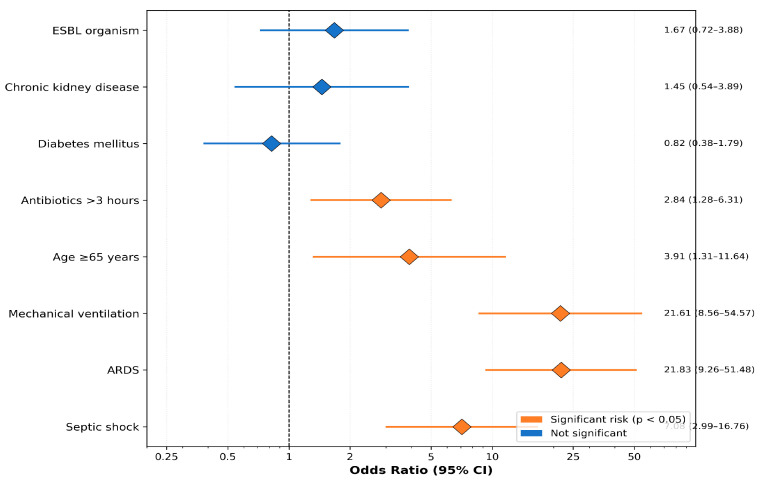
Forest plot of mortality risk factors showing odds ratios with 95% confidence intervals from univariate analysis.

Univariate logistic regression identified septic shock (OR: 7.08; 95% CI: 2.99–16.76), ARDS (OR: 21.83; 95% CI: 9.26–51.48), mechanical ventilation (OR: 21.61; 95% CI: 8.56–54.57), age ≥ 65 years (OR: 3.91; 95% CI: 1.31–11.64), and delayed antibiotics > 3 h (OR: 2.84; 95% CI: 1.28–6.31) as mortality risk factors (Table 6, Figure 4).

Multivariate logistic regression, adjusting for age, septic shock, mechanical ventilation, ARDS, and antibiotic timing, confirmed mechanical ventilation (aOR: 15.42; 95% CI: 5.67–41.95; *p* < 0.001) and septic shock (aOR: 5.23; 95% CI: 1.89–14.48; *p* = 0.001) as independent predictors of mortality. ARDS was no longer significant after adjustment, likely due to collinearity with mechanical ventilation. The Hosmer–Lemeshow test indicated adequate model fit (χ^2^ = 6.84, *p* = 0.554) (Table 7).

## 4. Discussion

This five-year analysis reveals distinct sepsis epidemiology in southwestern Saudi Arabia characterized by exceptionally high diabetes prevalence and UTI predominance. The 79.8% UTI predominance strikingly exceeds international patterns where respiratory infections typically lead (30–50% of cases [2,3]), other Saudi studies (Arabi et al.: ~15% [11]), and global estimates (12–15% [3]). To our knowledge, this represents the highest UTI prevalence reported in any sepsis cohort.

This exceptional UTI predominance almost certainly reflects our remarkable diabetes prevalence (71.5%), substantially exceeding both Arabi et al. (48.3%) and global sepsis population prevalence (~20% [14]). The diabetes–UTI–sepsis pathway is well established: glycosuria promotes bacterial proliferation, neutrophil function is impaired, and autonomic neuropathy causes incomplete bladder emptying [9,13,16]. Saudi Arabia ranks among countries with the highest diabetes prevalence globally [8], and our data suggest this metabolic disease burden substantially shapes regional sepsis epidemiology. This finding has important implications for empiric antibiotic selection in similar high-diabetes populations.

Our antimicrobial management data demonstrate median antibiotic timing of 1.8 h, with 24.7% achieving the one-hour target. While below optimal compliance rates reported in high-resource settings [17], this represents reasonable performance for our region. Notably, septic shock patients received antibiotics faster (1.4 vs. 1.9 h), suggesting appropriate triage prioritization. The high ESBL rate (34.8%) in our cohort supports the empiric use of piperacillin–tazobactam and carbapenems, as these agents provide reliable coverage against ESBL-producing organisms prevalent in our setting. Indeed, the 73.9% empiric coverage appropriateness rate may partly reflect this ESBL-informed prescribing strategy, as inadequate initial coverage of resistant uropathogens would have substantially reduced concordance rates. This observation aligns with regional antimicrobial resistance surveillance data supporting early broad-spectrum empiric coverage in populations with high ESBL prevalence, with subsequent de-escalation guided by culture results [18].

The significantly elevated creatinine and reduced platelets in septic shock align with established pathophysiology. Acute kidney injury in sepsis results from hemodynamic instability, inflammatory mediator exposure, and microcirculatory dysfunction [19]. Thrombocytopenia reflects coagulation cascade activation with platelet consumption [20]. These markers distinguished septic shock from sepsis in our cohort, supporting their utility for early severity recognition.

Multivariate analysis confirmed septic shock and mechanical ventilation as independent mortality predictors, consistent with international literature [21]. The loss of ARDS significance after multivariate adjustment likely reflects substantial collinearity with mechanical ventilation, as ARDS was the primary indication for intubation in this cohort. An interaction analysis between ARDS and mechanical ventilation did not reveal significant effect modification (*p* = 0.42 for interaction term), suggesting that the mortality risk attributed to mechanical ventilation is consistent regardless of ARDS status. We did not construct separate models excluding either variable due to the limited event count (31 deaths), which would risk overfitting. Notably, diabetes mellitus was not independently associated with mortality, possibly because UTI-source sepsis—predominant in diabetics—carries a more favorable prognosis than respiratory or abdominal sources. This paradox warrants further investigation in larger cohorts. Notably, recent population-based evidence suggests that preexisting diabetes may paradoxically confer lower adjusted mortality in sepsis, possibly through immune preconditioning mechanisms [15].

These findings carry several important clinical implications for practice in populations with high diabetes burden. First, the striking UTI predominance (79.8%) suggests that empiric sepsis management protocols in high-diabetes populations should prioritize early urine culture acquisition and empiric coverage targeting Gram-negative uropathogens. Second, the high ESBL rate (34.8%) supports initial broad-spectrum coverage with piperacillin–tazobactam or carbapenems, with planned de-escalation upon culture results. Third, the strong association between mechanical ventilation and mortality underscores the importance of avoiding unnecessary intubation through aggressive fluid resuscitation, early vasopressor initiation, and non-invasive ventilation where appropriate. Fourth, enhanced diabetes screening, glycemic monitoring, and UTI prevention strategies (including evaluation of SGLT2 inhibitor use) may reduce sepsis incidence in this high-risk population. Finally, our data support the implementation of structured sepsis bundles emphasizing the one-hour antibiotic target, particularly for septic shock patients where each hour of delay significantly impacts outcomes.

Our findings were contextualized within the existing literature. The 8.2% sepsis mortality and 38.7% septic shock mortality compare favorably with Arabi et al. (28.5% and 45.2%, respectively [11]). Recent Korean meta-analysis data report sepsis mortality of 28.9% [22], while a 2023 European multicenter study found septic shock mortality of 42.3% [23]. Our favorable outcomes likely reflect the predominance of UTI-derived sepsis, which carries better prognosis than respiratory sources given superior amenability to source control [18]. UTI-source sepsis demonstrates lower mortality than respiratory or abdominal sources across multiple studies, and our contemporary study period capturing recent SSC guideline implementation may also contribute. We acknowledge that comparison of crude mortality rates across studies with different case-mix and severity profiles has inherent limitations. Ideally, a standardized mortality ratio (SMR) based on validated prognostic scores such as APACHE II or SOFA would enable more robust benchmarking; however, inconsistent SOFA documentation in our retrospective data precluded this analysis. Future prospective studies should incorporate standardized severity scoring to facilitate meaningful cross-study comparisons.

This study has several important limitations. First, the single-center retrospective design limits generalizability to other Saudi or regional settings. Second, potential selection bias may arise from the exclusion of patients with incomplete documentation (8.7% of screened admissions); however, the missing data were predominantly related to absent SOFA scoring rather than systematic exclusion of particular patient subgroups. Third, inconsistent SOFA documentation precluded severity-stratified analysis and calculation of standardized mortality ratios. Fourth, the observational nature precludes causal inference regarding antibiotic timing effects. Fifth, the study period (2020–2024) overlapped with the COVID-19 pandemic, which may have influenced ICU admission patterns and outcomes [24]. While respiratory infections constituted only 14.1% of sepsis sources in our cohort, we were unable to systematically assess COVID-19 co-infection status or determine whether the pandemic period influenced the relative distribution of sepsis etiologies. Sixth, detailed data on diabetes management (insulin vs. oral hypoglycemic agents including SGLT2 inhibitors, which are known to increase UTI risk) were not systematically captured, precluding analysis of the impact of specific antidiabetic regimens on infection susceptibility. Seventh, gender-stratified mortality analysis was not performed, though this represents an important future direction given known sex differences in UTI susceptibility and sepsis outcomes. Eighth, data on antibiotic regimen adjustments following culture and susceptibility results were not systematically collected, limiting assessment of de-escalation practices. Ninth, advanced model validation techniques (bootstrapping, split-sample) were not performed due to sample size constraints. Tenth, peak laboratory values were used as they represent maximum physiological derangement, but the timing of these peaks relative to sepsis diagnosis was not uniformly captured. Future multicenter prospective studies with standardized severity scoring, comprehensive diabetes phenotyping, and longitudinal antimicrobial stewardship data should address these limitations.

## 5. Conclusions

This five-year retrospective study demonstrates that high diabetes prevalence (71.5%) shapes a distinct regional sepsis epidemiology in southwestern Saudi Arabia, characterized by unprecedented UTI predominance (79.8%) and favorable mortality compared with national and international benchmarks. Septic shock and mechanical ventilation were confirmed as independent mortality predictors. Clinically, these findings support region-specific sepsis management protocols emphasizing (1) early urine culture acquisition and Gram-negative-focused empiric coverage; (2) broad-spectrum antimicrobial selection informed by high local ESBL rates; (3) early recognition and aggressive management of septic shock; and (4) integrated diabetes care with attention to UTI prevention, including review of SGLT2 inhibitor prescribing in high-risk patients. Future multicenter prospective studies with standardized severity scoring and comprehensive diabetes phenotyping are needed to validate these findings and develop population-specific sepsis management algorithms.

## Figures and Tables

**Table 1:** Baseline demographic and clinical characteristics.

Characteristic	Total (n = 263)	Sepsis (n = 232)	Septic Shock (n = 31)	*p*-Value
Age, years, median (IQR)	73 (55–84)	73 (56–84)	70 (41–84)	0.930
Male sex, n (%)	153 (58.4)	138 (59.7)	15 (48.4)	0.312
Saudi nationality, n (%)	253 (96.2)	223 (96.1)	30 (96.8)	1.000
Diabetes mellitus, n (%)	188 (71.5)	167 (72.0)	21 (67.7)	0.621
Hypertension, n (%)	173 (65.8)	154 (66.4)	19 (61.3)	0.573
Chronic kidney disease, n (%)	36 (13.7)	31 (13.4)	5 (16.1)	0.675
Coronary artery disease, n (%)	31 (11.8)	27 (11.6)	4 (12.9)	0.769
Medical admission, n (%)	166 (75.8)	147 (76.2)	19 (73.1)	0.718

IQR: interquartile range.

**Table 2:** Sources of infection and isolated organisms.

Variable	n	%
Source of Infection		
Urinary tract	210	79.8
Pulmonary	37	14.1
Abdominal	7	2.7
Other/Unknown	9	3.4
Isolated Organisms (n = 161)		
Escherichia coli	69	42.9
Klebsiella pneumoniae	45	28.0
Enterococcus species	22	13.7
Proteus species	14	8.7
Pseudomonas aeruginosa	11	6.8
ESBL-producing Enterobacteriaceae	56	34.8

ESBL: extended-spectrum beta-lactamase. Organism percentages calculated from culture-positive cases (n = 161).

**Table 3:** Antimicrobial management characteristics.

Variable	Total (n = 263)	Sepsis (n = 232)	Septic Shock (n = 31)	*p*-Value
Time to antibiotics, hours, median (IQR)	1.8 (1.1–2.9)	1.9 (1.2–3.1)	1.4 (0.8–2.1)	0.038
Antibiotics within 1 h, n (%)	65 (24.7)	53 (22.8)	12 (38.4)	0.063
Antibiotics within 3 h, n (%)	198 (75.3)	172 (74.1)	26 (83.9)	0.279
Empiric regimen, n (%)				
Piperacillin–tazobactam	115 (43.7)	98 (42.2)	17 (54.8)	0.219
Meropenem	78 (29.7)	68 (29.3)	10 (32.3)	0.834
Ceftriaxone	42 (16.0)	40 (17.2)	2 (6.5)	0.137
Other	28 (10.6)	26 (11.2)	2 (6.5)	0.547
Appropriate empiric coverage, n/N (%)	119/161 (73.9)	104/140 (74.3)	15/21 (71.4)	0.786

IQR: interquartile range. Appropriate coverage assessed in culture-positive cases only.

**Table 4:** Laboratory findings stratified by diagnosis.

Parameter	Sepsis (n = 232)	Septic Shock (n = 31)	*p*-Value
Peak creatinine, mg/dL	1.08 (0.75–1.81)	1.65 (0.92–2.50)	0.026
Peak WBC, ×10^3^/μL	8.60 (6.37–11.55)	9.33 (6.83–14.68)	0.178
Serum lactate, mmol/L	1.15 (0.68–1.98)	1.58 (0.82–2.85)	0.089
Peak glucose, mg/dL	133 (97–188)	129 (87–185)	0.527
Peak platelet, ×10^3^/μL	271 (197–369)	194 (112–323)	0.030
Peak CRP, mg/L	87 (44–152)	98 (52–178)	0.312
Peak bilirubin, mg/dL	0.8 (0.5–1.4)	1.1 (0.6–2.3)	0.074

Values: median (IQR). WBC: white blood cell; CRP: C-reactive protein.

**Table 5:** Interventions and clinical outcomes.

Variable	Sepsis (n = 232)	Septic Shock (n = 31)	*p*-Value
Mechanical ventilation, n (%)	50 (21.6)	21 (67.7)	<0.001
Vasopressors, n (%)	0 (0)	31 (100)	<0.001
ECMO, n (%)	16 (6.9)	9 (29.0)	<0.001
CRRT, n (%)	13 (5.6)	4 (12.9)	0.155
ARDS, n (%)	37 (16.0)	13 (41.9)	<0.001
ICU LOS, days, median (IQR)	6 (4–10)	9 (5–16)	0.018
In-hospital mortality, n (%)	19 (8.2)	12 (38.7)	<0.001
30-day mortality, n/N (%) *	21/203 (10.3)	11/24 (45.8)	<0.001

ECMO: extracorporeal membrane oxygenation; CRRT: continuous renal replacement therapy; ARDS: acute respiratory distress syndrome; ICU LOS: intensive care unit length of stay. * Available for 227 patients (86.3%).

**Table 6:** Univariate analysis of risk factors for in-hospital mortality.

Risk Factor	OR	95% CI	*p*-Value
Septic shock (vs. sepsis)	7.08	2.99–16.76	<0.001
ARDS	21.83	9.26–51.48	<0.001
Mechanical ventilation	21.61	8.56–54.57	<0.001
Age ≥ 65 years	3.91	1.31–11.64	0.014
Antibiotics > 3 h	2.84	1.28–6.31	0.010
Diabetes mellitus	0.82	0.38–1.79	0.621
Chronic kidney disease	1.45	0.54–3.89	0.462
ESBL organism	1.67	0.72–3.88	0.234

OR: odds ratio; CI: confidence interval; ARDS: acute respiratory distress syndrome; ESBL: extended-spectrum beta-lactamase.

**Table 7:** Multivariate logistic regression for in-hospital mortality.

Variable	aOR	95% CI	*p*-Value
Septic shock	5.23	1.89–14.48	0.001
Mechanical ventilation	15.42	5.67–41.95	<0.001
Age ≥ 65 years	2.58	0.79–8.43	0.117
Antibiotics > 3 h	1.89	0.76–4.71	0.172
ARDS	2.14	0.68–6.73	0.193

aOR: adjusted odds ratio; CI: confidence interval. Hosmer–Lemeshow test: χ^2^ = 6.84, *p* = 0.554.

## Data Availability

The data presented in this study are available on reasonable request from the corresponding author. The data are not publicly available due to privacy restrictions.

## Supplementary Materials

The following supporting information can be downloaded at: https://www.mdpi.com/article/10.3390/healthcare14050680/s1, Supplementary File S1: STROBE Statement—Checklist of Items That Should Be Included in Reports of Cohort Studies.
